# *Candidatus* Anthektikosiphon siderophilum OHK22, a New Member of the Chloroflexi Family Herpetosiphonaceae from Oku-okuhachikurou Onsen

**DOI:** 10.1264/jsme2.ME20030

**Published:** 2020-07-29

**Authors:** Lewis M Ward, Woodward W Fischer, Shawn E McGlynn

**Affiliations:** 1 Department of Earth & Planetary Sciences, Harvard University, Cambridge, MA USA; 2 Earth-Life Science Institute, Tokyo Institute of Technology, Tokyo, Japan; 3 Division of Geological & Planetary Sciences, California Institute of Technology, Pasadena, CA, USA

**Keywords:** chloroflexota, *Herpetosiphon*, predatory bacteria, aerobic respiration, metagenomics

## Abstract

We report the draft metagenome-assembled genome of a member of the Chloroflexi family Herpetosiphonaceae from microbial biofilms developed in a circumneutral, iron-rich hot spring in Japan. This taxon represents a novel genus and species—here proposed as *Candidatus* Anthektikosiphon siderophilum—that expands the known taxonomic and genetic diversity of the Herpetosiphonaceae and helps orient the evolutionary history of key traits like photosynthesis and aerobic respiration in the Chloroflexi.

The Chloroflexi family Herpetosiphonaceae is made up of aerobic, nonphototrophic filamentous bacteria, and is the sister group to the clade of well known, photosynthetic Chloroflexia that includes the genera *Roseiflexus* and *Chloroflexus* (Kiss *et al.*, 2011; Ward *et al.*, 2015; Ward *et al.*, 2018a). The Herpetosiphonaceae family is best known for the predatory bacteria of the genus *Herpetosiphon* (*e.g.* Castenholz, 2012), but the ecological and physiological diversity of this family is not yet well constrained. Deeper sampling of the metabolic and genetic diversity of the Herpetosiphonaceae is critical for understanding the evolution of aerobic respiration, phototrophy, and carbon fixation (variably via the Calvin cycle and 3-hydroxyproprionate bicycle) within the Chloroflexi—by orienting the relative order and timing of acquisition of these traits within this group (*e.g.* Shih *et al.*, 2017; Ward *et al.*, 2018a). We discovered a draft genome of a novel genus in the Herpetosiphonaceae recovered from genome-resolved metagenomic sequencing of samples of mineralized biofilms from Okuoku-hachikurou Onsen—a carbonate-forming, iron-rich hot spring in Akita Prefecture, Japan, that shares some mineralogical features with iron formation deposits observed in the early parts of the sedimentary record (Takashima *et al.*, 2011; Ward *et al.*, 2017a). This taxon provided valuable insight into the aerobic history of the Chloroflexia and expanded the known diversity of Herpetosiphonaceae.

OHK22 was recovered from genome-resolved metagenomic sequencing of samples from Okuoku-hachikurou Onsen (OHK) in Akita Prefecture, Japan. The geochemistry and microbial diversity and ecology of OHK has been characterized previously (Takashima *et al.*, 2011; Ward *et al.*, 2017a). In brief, OHK is an iron-carbonate hot spring in Akita Prefecture, Japan, with source waters supersaturated in CO_2_, no detectable dissolved oxygen, pH 6.8, ~45°C, and containing 114‍ ‍μM dissolved Fe^2+^ (Ward *et al.*, 2017a). As the spring water flows downstream, the water cools, exchanges gases with the atmosphere, and the ferrous iron is oxidized and deposited forming mixed aragonite-iron oxide laaminated rocks. Samples for metagenomics were collected in September 2016 from the “Shallow Source” and “Canal” sites described in Ward *et al.*, 2017a (Fig. 1). Thin biofilms (<1‍ ‍mm-thick) were scraped from mineral precipitates using sterile forceps and spatulas (~0.25‍ ‍cm^3^ of material). Cells were lysed and DNA preserved in the field using a Zymo Terralyzer BashingBead Matrix and Xpedition Lysis Buffer (Zymo Research). Cells were disrupted immediately by attaching the polyethylene sample tubes to the blade of a cordless reciprocating saw (Makita JR101DZ), and operating for 1‍ ‍min. Following return to the lab, microbial DNA was extracted and purified with a Zymo Soil/Fecal DNA extraction kit (Zymo Research). DNA was quantified with a Qubit 3.0 fluorimeter (Life Technologies) according to manufacturer’s instructions following DNA extraction. Purified DNA was submitted to SeqMatic LLC (Fremont) for library preparation sequencing. Library preparation was performed using an Illumina Nextera XT DNA Library Preparation Kit per the manufacturer’s protocols, producing constructs with an average size of ~519 bp prior to 2×100 bp paired-end sequencing via Illumina HiSeq 4000 technology. Raw sequence reads were preprocessed and quality controlled (adapter trimming, contaminant filtering, and merging of paired reads) with BBTools (Bushnell, 2014) prior to coassembling the two samples with MegaHit v. 1.02 (Li *et al.*, 2016) and genome bins constructed based on differential coverage using MetaBAT (Kang *et al.*, 2015), MaxBin (Wu *et al.*, 2014), and CONCOCT (Alneberg *et al.* 2013. CONCOCT: clustering contigs on coverage and composition. arXiv preprint arXiv:1312.4038.) followed by replication and refinement by DAS Tool (Sieber *et al.*, 2018). Completeness and contamination/redundancy were determined with CheckM v1.1.2 using the bacterial marker set UID1452 (Parks *et al.*, 2015). The genome was uploaded to RAST v2.0 for annotation and characterization (Aziz *et al.*, 2008). Presence or absence of metabolic pathways of interest was predicted using MetaPOAP v1.0 (Ward *et al.*, 2018b). Taxonomic assignment was determined with GTDB-Tk v0.3.2 (Parks *et al.*, 2018). Genomes were compared with AAI (Rodriguez-R and Konstantinidis, 2014) to verify species and genus-level divisions. Organismal phylogenies were built using concatenated ribosomal proteins following methods from Hug *et al.* (2016). Protein sequences were extracted from genomes using the *tblastn* function of BLAST+ (Camacho *et al.*, 2009) and aligned using MUSCLE (Edgar, 2004). Trees were calculated using RAxML v8.2.12 (Stamatakis, 2014) on the Cipres science gateway (Miller *et al.*, 2010). Transfer bootstrap support values were calculated by BOOSTER (Lemoine *et al.*, 2018), and trees were visualized with the Interactive Tree of Life viewer (Letunic and Bork, 2016). Presence of genes associated with iron cycling was determined with FeGenie (Garber *et al.*, 2020). Comparison of genome contents was made based on annotations in RAST (Aziz *et al.*, 2008) together with analyses by OrthoVenn2 (Xu *et al.*, 2019) and KEGG-decoder (Graham *et al.*, 2018). All software was run with default parameters; further testing and optimization of parameters for each computational analysis may allow future improvement of assembly and MAG quality.

Metagenomic sequencing was performed on biofilm and mineralized samples from the “Shallow Source” and “Canal” sites at OHK Onsen (Fig. 1) (Ward *et al.*, 2017a). Both samples consisted of layered iron oxide and calcium carbonate travertine with very thin (<–1‍ ‍mm) biofilms submerged in flowing hot spring water ~44°C with relatively low dissolved O_2_ (<62‍ ‍μM) and high dissolved ferrous iron (>83‍ ‍μM). Previous work has suggested that the microbial community in this environment is capable of only limited rates of primary productivity and organic carbon accumulation and is fueled primarily by iron oxidation and aerobic respiration with only small contributions from photosynthesis (Ward *et al.*, 2017a).

Metagenomic sequencing of these two samples from OHK Onsen resulted in 52 GB of raw sequence data which produced a 579‍ ‍Mb coassembly with an N50 of 27,219 nt. Binning of this metagenome assembly to produce metagenome-assembled genomes (MAGs) produced a number of high quality genomes following current standards (Bowers *et al.*, 2017) including OHK22 described here and OHK40 which has been described elsewhere (Ward *et al.* 2019. Genomic evidence for phototrophic oxidation of small alkanes in a member of the chloroflexi phylum. *bioRxiv* 531582.). Reads mapped to the OHK22 MAG were recovered with an average of 5-fold coverage from the “Canal” metagenome and 48-fold coverage from the “Shallow Source” metagenome. The OHK22 genome is 6,335,032 nt and 62.48% GC, and was recovered as 285 contigs with an N50 of 33,814 and a minimum contig length of 2,609 nt. The genome was estimated to be 96.97% complete with 0.91% contamination/redundancy and 0% strain heterogeneity. The genome contained 45 tRNAs and 5,771 coding sequences. We determined that OHK22 belonged to the Chloroflexi family Herpetosiphonaceae via analysis with concatenated ribosomal protein phylogenies and with GTDB-Tk, but results indicated that this taxon could not be assigned to any previously described genus in this family (Fig. 2).

A 16S rRNA sequence was not recovered in the OHK22 MAG; however, our initial 16S amplicon sequencing survey from the same sites (Ward *et al.*, 2017a) revealed the presence of three closely related Herpetosiphonaceae OTUs at OHK with total relative abundance at the “Canal” and “Shallow Source” sites of ~0.3%. Estimates from the later acquired samples which are the focus of this study are consistent with this: the relative abundance of OHK22 in the microbial community based on the proportion of raw metagenomic reads and the coassembly that map onto the OHK22 genome suggest that this organism makes up no more than ~1% of the sequenced microbial community.

The previously characterized genomic diversity of the Herpetosiphonaceae consisted of *Kallotenue papyrolyticum* (Hedlund *et al.*, 2015) and three species within the genus *Herpetosiphon* (*H. aurantiacus*, Kiss *et al.*, 2011; *H. geysericola*, Ward *et al.*, 2015; and *H. llansteffanense*, Livingstone *et al.*, 2018). Other *Herpetosiphon* strains have been isolated including members of the species *H. gulosus* and *H. giganteus* (Pan *et al.*, 2017), but to our knowledge genome sequences are not yet available for these organisms. Phylogenetic analyses (Fig. 2) and classification via GTDB-Tk placed OHK22 on a branch between *Herpetosiphon* and *Kallotenue*. AAI analyses (Rodriguez-R and Konstantinidis, 2014) displayed <60% similarity between OHK22 and other Herpetosiphonaceae—a level consistent with genus-level divergence. A 16S rRNA sequence was not recovered in the OHK22 MAG; however, 16S amplicon sequencing of the same samples (Ward *et al.*, 2017a) revealed the presence of a three closely related OTUs of Herpetosiphonaceae that likely represent OHK22 and/or closely related strains. These OTUs displayed ~86% similarity to *Herpetosiphon aurantiacus*, again consistent with genus-level divergence. Sequences >90% similar to these OHK Herpetosiphonaceae OTUs have been recovered from other harsh environments, particularly cold desert soils (*e.g.* Sattin *et al.*, 2009; Borin *et al.*, 2010; Mapelli *et al.* 2012. Succession of a bacterial rhizospheric community along a chronosequence of a cold desert. In *BIODESERT International Workshop-Microbial Diversity in Desert Extreme Environment “Microarrays from theory to application”*). This pattern implies that there is a broad environmental distribution for this genus-level clade in regions with low nutrient availability and high osmotic and/or temperature stress.

While members of the Chloroflexi are best known from thermophilic environments like hot springs (*e.g.* Klatt *et al.*, 2013; Thiel *et al.*, 2018), much of the known diversity of this phylum was derived from mesophilic or even psychrophilic environments (*e.g.* Inagaki *et al.*, 2003; Hug *et al.*, 2013; Mehrshad *et al.*, 2018). Within the Herpetosiphonaceae, only the basal lineage *Kallotenue papyrolyticum* has previously been shown to thrive under high temperatures >40°C (Table 1). OHK22 was recovered from a hot spring that maintains a temperature of ~44°C, and therefore expanded the known diversity of thermophilic Herpetosiphonaceae.

While prediction of function from protein sequences alone are tentative in the absence of isolate-based experimental verification, we found that the metabolic pathways encoded in the OHK22 genome were broadly similar to that of other members of the Herpetosiphonaceae (Kiss *et al.*, 2011; Hedlund *et al.*, 2015; Ward *et al.*, 2015; Livingstone *et al.*, 2018); these are consistent with a lifestyle as an aerobic heterotroph (Fig. 3). OHK22 included genes for a high-potential electron transport chain feeding aerobic respiration (*i.e.* a *bc*-type Complex III and an A-family heme copper O_2_ reductase). The OHK22 MAG did not recover genes associated with denitrification (*e.g.* *nirS*, *nirK*, *qNor*, *cNor*), sulfate reduction (*e.g.* *aprA*, *aprB*, *dsrA*, *dsrB*), carbon fixation (*e.g.* rubisco, ATP-citrate lyase, or malyl-CoA lyase), nitrogen fixation (*e.g.* *nifHDK*), or phototrophy (*e.g.* *pufL*, *pufM*, *bchX*, *bchY*, *bchZ*). Given the high completeness of the OHK22 MAG, MetaPOAP estimated the likelihood of False Negatives for the presence of these pathways to be very low (*e.g.* ~7×10^–10^ for phototrophy based on the absence of the key marker genes *pufL*, *pufM*, *bchX*, *bchY*, and *bchZ*). This provides confidence that these pathways are actually absent in OHK22. Analysis of the genome with CXXCH_finder (McGlynn *et al.*, 2015) revealed that no proteins encoded by OHK22 have more than 2 heme-binding domains, suggesting that despite the iron oxide-rich environment this organism is not capable of dissimilatory iron reduction (or oxidation) via extracellular electron transfer mechanisms. FeGenie found fewer genes associated with siderophore synthesis, iron transport, iron regulation, and iron storage in OHK22 as compared to other Herpetosiphonaceae (*e.g.* 4 genes for iron transport in OHK22 versus 17 in *Herpetosiphon aurantiacus* and 11 in *Kallotenue papyrolyticum*, Supplemental Table S1). This may reflect adaptation of OHK22 to an iron-rich environment in which less energetic investment in iron acquisition is necessary as compared to the relatively iron-poor environments from which other Herpetosiphonaceae have been recovered. FeGenie identified one protein encoded by OHK22 similar to sulfocyanin (SoxE) which has been implicated in iron oxidation in some acidophilic iron oxiders (*e.g.* Castelle *et al.*, 2015) but no other proteins related to iron oxidation or reduction.

OHK22 lacks a B-family heme copper O_2_ reductase complex that is found in a number of thermophilic Chloroflexi (including phototrophs and nonphototrophs from the classes Anaerolineae, Ardenticatenia, and Chloroflexia, *e.g.* Hemp *et al.*, 2015; Ward *et al.*, 2019a). The distribution of B-family heme copper O_2_ reductases in Chloroflexi is unlike that of A-family heme-copper O_2_ reductases, the latter of which appears to be ancestral in many class-level Chloroflexi clades; instead, the B-family heme-copper O_2_ reductases appear to be derived traits that have been acquired independently through horizontal gene transfer in certain Chloroflexi lineages (Ward *et al.*, 2018a). The absence of B-family heme copper O_2_ reductases, Alternative Complex III, phototrophy, and carbon fixation in OHK22 indicate that the ancestral electron transport chains of Chloroflexia most likely contained high-potential metabolism built around A-family heme-copper O_2_ reductases and *b*c-type Complex III. This further highlights the rich and delayed history of phototrophy relative to aerobic respiration within the Chloroflexia (Ward *et al.*, 2015; Shih *et al.*, 2017; Ward *et al.*, 2018a).

Genomic comparisons using OrthoVenn2 (Xu *et al.*, 2019) revealed 331 protein sequences in 117 clusters encoded in OHK22 genome that were not present in other Herpetosiphonaceae. These primarily consisted of short, incomplete sequences and/or hypothetical proteins of unknown function. Further examination of the suite of genes present in OHK22 but not Herpetosiphonaceae via analyses from RAST and KEGG-decoder revealed that OHK22 possessed additional genes associated with metal tolerance and transport of metals other than iron. These included protein-coding genes annotated as a P-type Mg^2+^ transport ATPase, the Fe-Mn transporter MntH, and magnesium and cobalt efflux protein CorC. The relative enrichment of metal tolerance and transport genes in this taxon may reflect adaptation to the metal-rich environment in which OHK22 lives.

While it is not currently possible to determine whether or not OHK22 is a predatory bacterium like members of *Herpetosiphon* in the absence of an enrichment or isolate, we found that OHK22 lacks many genes found in members of *Herpetosiphon* that have been associated with predatory activity. These include hydroxymethyl glutaryl CoA lyase, mevalonate kinase, NADPH-dependent flavin mononucleotide (FMN) reductase, tryptophan 2,3-dioxygenase, serine protease, and von Willebrand factor (Livingstone *et al.*, 2018). However, OHK22 did encode proteins for degrading extracellular polymers including chitinase, glucoamylase, and multiple peptidoglycan hydrolases. It may be that OHK22 fills an ecological niche more similar to *Kallotenue* (Cole *et al.*, 2013) than to *Herpetosiphon* (Lewin, 1970)—as a nonpredatory aerobic heterotroph targeting extracellular organic carbon sources such as polysaccharides produced by other microbes in the environment.

While a variety of carotenoid pigments are known to be synthesized by many Chloroflexia, including members of the Herpetosiphonaceae (*e.g.* *H. aurantiacus*, Kiss *et al.*, 2011), the OHK22 MAG did not contain downstream components of carotenoid synthesis pathways found in closely related organisms. The OHK22 genome encoded proteins such as phytoene synthase (CrtB) and phytoene desaturase (CrtI), but lacked lycopene cyclase (CruA), γ-carotene 1′,2′-hydratase (CruF), and other downstream carotenoid modification proteins seen in other Herpetosiphonaceae genomes (Kiss *et al.*, 2011; Hedlund *et al.*, 2015; Ward *et al.*, 2015; Livingstone *et al.*, 2018). It therefore seems likely OHK22 is capable of synthesizing lycopene but lacks the ability to produce more complex carotenoids.

The OHK22 genome did not encode a flagellum and its mode of motility is uncertain. Because genetic markers for the gliding motility of other Herpetosiphonaceae have not yet been identified (*e.g.* Kiss *et al.*, 2011), it cannot be determined at this time whether or not OHK22 is a motile organism. Like other Chloroflexi (*e.g.* Sutcliffe, 2011; Pace *et al.*, 2015; Ward *et al.*, 2018a)—but unlike the closely related phyla Armatimonadetes and *Ca.* Eremiobacterota (WPS-2) (Ward *et al.*, 2017b, 2019b)—OHK22 does not encode LPS biosynthesis or outer membrane proteins. This supports the hypothesis of a monoderm nature for the Chloroflexi membrane (Sutcliffe, 2010, 2011); current relationships support a derived, secondary loss of the outer membrane from a diderm last common ancestor of Chloroflexi, Armatimonadetes, and *Ca.* Eremiobacterota (Ward *et al.*, 2019b).

## Conclusions

The environment and apparent ecological niche, genomic content (*e.g.* number of coding sequences, GC content), and putative metabolic capabilities of OHK22 suggest that this organism may reflect a transitional form of the Herpetosiphonaceae between ancestral high temperature polysaccharide degraders like *Kallotenue* and more derived low temperature predatory bacteria like *Herpetosiphon*. Together with this evidence for the evolutionary and ecological divergence of OHK22 from the described genera of Herpetosiphonaceae, accepted metrics for genome-based taxonomy indicate that OHK22 represents a novel genus-level lineage. We therefore proposed the assignment of OHK22 to a novel candidate genus and species within the Herpetosiphonaceae, with the name *Candidatus* Anthektikosiphon (from the Greek *anthektikós*, durable or tough, and *siphon*, tube or pipe, in reference to the apparently typical extreme habitats of these organisms and the filamentous morphology typical of Chloroflexia) siderophilum (from the Greek *sideros*, iron, and *philos*, loving, in reference to the high iron adaptation of this species). We propose *Ca.* Anthektikosiphon siderophilum OHK22 as the type genome for the genus and species, with official classification pending isolation and characterization of at least one strain of these taxa.

Molecular clock studies estimated that *Kallotenue* and *Herpetosiphon* lineages diverged from each other—and from other members of Chloroflexia (*i.e.* the phototrophic families Chloroflexaceae and Roseiflexaceae)— approximately 1.1–1.3 billion years ago, followed by divergence of other genus-level lineages of Chloroflexia >400 million years ago (Shih *et al.*, 2017). If those estimates are even broadly correct, it is reasonable to predict that *Ca.* Anthektikosiphon diverged from other Herpetosiphonaceae lineages roughly several hundred million years ago. Given recent estimates for the rate of diversification of bacterial lineages over geological time (Louca *et al.*, 2018) it is expected that many more lineages of *Ca.* Anthektikosiphon will have arisen in this time. While some of these lineages may have subsequently gone extinct, it seems likely that thorough sampling of appropriate environments will yield discovery of more extant diversity of *Ca.* Anthektikosiphon and other related lineages.

## Data availability

This Whole Genome Shotgun project has been deposited at DDBJ/ENA/GenBank under the accession JABXJS000000000. The version described in this paper is version JABXJS010000000.

## Citation

Ward, L. M., Fischer, W. W., and McGlynn, S. E. (2020) *Candidatus* Anthektikosiphon siderophilum OHK22, a New Member of the Chloroflexi Family Herpetosiphonaceae from Oku-okuhachikurou Onsen. *Microbes Environ ***35**: ME20030.

https://doi.org/10.1264/jsme2.ME20030

## Supplementary Material

Supplementary Material 1

Supplementary Material 2

Supplementary Material 3

## Figures and Tables

**Fig. 1.**
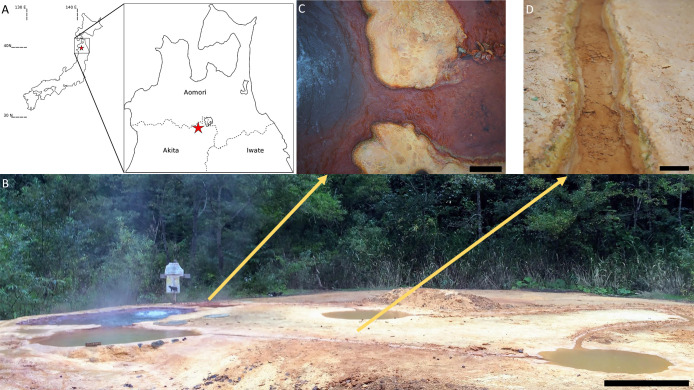
Oku-okuhachikurou Onsen (OHK), Japan, and the source of *Ca.* Anthektikosiphon siderophilum. A: Overview map of Japan showing the location of OHK in Akita Prefecture. Modified from Ward *et al.* (2017a). B: Panorama of OHK pools and channels at the time of sampling. Source pool occurs at the left, with flow downstream to the right. Scale bar ~1 m. Samples were collected from the “Shallow Source” (C, scale bar ~‍ ‍30‍ ‍cm) and “Canal” (D, scale bar ~10‍ ‍cm) sites as scrapings of thin green-colored biofilms overlying iron-oxide and aragonite travertine along the water line.

**Fig. 2.**
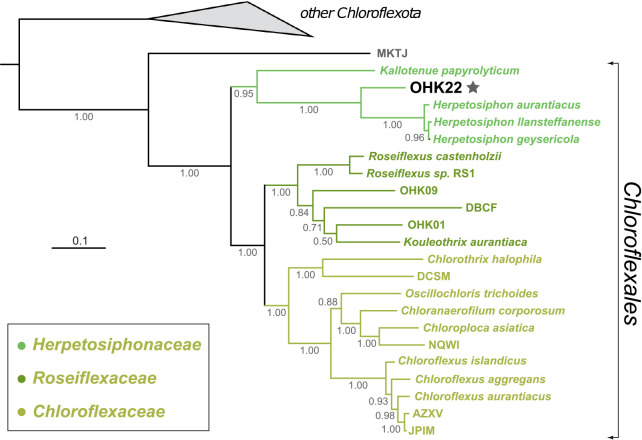
Phylogeny of the Chloroflexia built with concatenated ribosomal protein sequences and labeled with family- and order-level taxonomy as determined by GTDB-tk. OHK22 (marked with star) branches between *Herpetosiphon* and *Kallotenue* within the Herpetosiphonaceae family. For metagenome-assembled genomes without species or strain names, leaves are labeled with NCBI WGS database IDs.

**Fig. 3.**
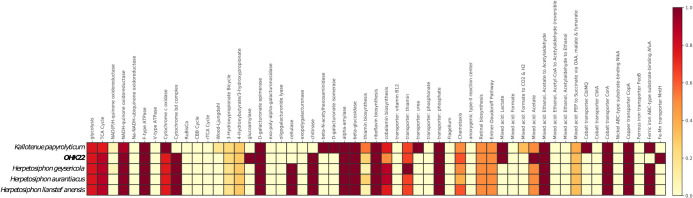
KEGG-decoder heatmap of relative abundance of functional genes between OHK22 and previously described genomes of Herpetosiphonaceae.

**Table 1.** Herpetosiphonaceae genomes compared in this study.

MAG Id	GTDB-Tk taxonomy	Source environment	Source and/or optimal growth T	Reference	NCBI WGS accession number	Genbank assembly accession	Completeness	Contamination	Strain heterogeneity	Contigs	Size (Mb)	GC%	Coding sequences
OHK22	d__Bacteria; p__Chloroflexota; c__Chloroflexia; o__Chloroflexales; f__Herpetosiphonaceae; g__;s__	Iron-rich hot spring	Source at 44°C	This study	JABXJS		96.97	0.91	0	285	6.35	62.48	5131
Kallotenue papyrolyticum	d__Bacteria; p__Chloroflexota; c__Chloroflexia; o__Chloroflexales; f__Herpetosiphonaceae; g__JKG1; s__JKG1 sp000526415	cellulolytic enrichment in Great Boiling Spring, Nevada	Optimum 55°C	Cole *et al.*, 2013; Hedlund *et al.*, 2015	JAGA	GCA_000526415.1	97.58	0	0	4	4.475263	65.77	3948
Herpetosiphon aurantiacus	d__Bacteria; p__Chloroflexota; c__Chloroflexia; o__Chloroflexales; f__Herpetosiphonaceae; g__Herpetosiphon; s__Herpetosiphon aurantiacus	Slimy coating of a freshwater alga		Holt and Lewin, 1968; Kiss *et al.*, 2011	N/A	GCA_000018565.1	98.18	1.82	0	3	6.346587	50.9	5617
Herpetosiphon geysericola	d__Bacteria; p__Chloroflexota; c__Chloroflexia; o__Chloroflexales; f__Herpetosiphonaceae; g__Herpetosiphon; s__Herpetosiphon geysericola	Biofilm adjacent to a hot springin Baja California, Mexico	Maximum T of 38–40°C	Lewin, 1970; Ward *et al.*, 2015	LGKP	GCA_001306135.1	99.09	0.91	0	46	6.140412	50.75	5445
Herpetosiphon llanstefanensis	d__Bacteria; p__Chloroflexota; c__Chloroflexia; o__Chloroflexales; f__Herpetosiphonaceae; g__Herpetosiphon; s__Herpetosiphon‍ ‍sp003205875	Stream-edge soil	Better growth at 30°C than 37°C or 42°C	Livingstone *et al.*, 2018	PUBZ	GCA_003205875.1	99.09	1.82	0	169	6.140145	50.77	5401
