# Supplementary material for: Candidatus Anthektikosiphon siderophilum OHK22, a New Member of the Chloroflexi Family Herpetosiphonaceae from Oku-okuhachikurou Onsen
Source: Microbes Environ. 2020 Jul 29;35(3):ME20030. doi: 10.1264/jsme2.ME20030 (PMC7511795; doi:10.1264/jsme2.ME20030)
Supplement: Supplementary file 3 — Supplementary Material 3 [file 35_20030_s3.pdf]

Supplemental Table 1: Number of proteins encoded by Herpetosiphonaceae genomes associated with iron acquisition and metabolism as determined by FeGenie.

Supplemental Table 2: Annotated proteins encoded in the OHK22 genome as determined by RAST.
